# LAMR1 restricts Zika virus infection by attenuating the envelope protein ubiquitination

**DOI:** 10.1080/21505594.2021.1948261

**Published:** 2021-07-20

**Authors:** Dingwen Hu, Yingchong Wang, Aixin Li, Qin Li, Caifeng Wu, Muhammad Adnan Shereen, Shanyu Huang, Kailang Wu, Ying Zhu, Wenbiao Wang, Jianguo Wu

**Affiliations:** a State Key Laboratory of Virology, College of Life Sciences, Wuhan University, Wuhan China; b Guangdong Provincial Key Laboratory of Virology, Institute of Medical Microbiology, Jinan University, Guangzhou China; c Department of Medicine & Therapeutics, Li Ka Shing Institute of Health Sciences, Lui Che Woo Institute of Innovative Medicine, the Chinese University of Hong Kong, Sha Tin, Hong Kong, China; d Foshan Institute of Medical Microbiology, Foshan China

**Keywords:** Laminin receptor 1, LAMR1, Zika virus, ZIKV, E protein, ubiquitination, eukaryotic translation initiation factor 3 subunit 5, EIF3S5

## Abstract

Zika virus (ZIKV) infection can cause severe neurological disorders, including Guillain–Barre syndrome and meningoencephalitis in adults and microcephaly in fetuses. Here, we reveal that laminin receptor 1 (LAMR1) is a novel host resistance factor against ZIKV infection. Mechanistically, we found that LAMR1 binds to ZIKV envelope (E) protein *via* its intracellular region and attenuates E protein ubiquitination through recruiting the deubiquitinase eukaryotic translation initiation factor 3 subunit 5 (EIF3S5). We further found that the conserved G282 residue of E protein is essential for its interaction with LAMR1. Moreover, a G282A substitution abolished the binding of E protein to LAMR1 and inhibited LAMR1-mediated E protein deubiquitination. Together, our results indicated that LAMR1 represses ZIKV infection through binding to E protein and attenuating its ubiquitination.

## Introduction

Zika virus (ZIKV) is a mosquito-borne virus belonging to the genus Flavivirus within the family *Flaviviridae*, which also includes Japanese encephalitis virus (JEV), West Nile virus (WNV), and Dengue virus (DENV) [1]. ZIKV was originally isolated in 1947 in Uganda from a caged febrile Rhesus macaque [2]. The first known ZIKV outbreak occurred in 2007 in Yap Island, Federated States of Micronesia [3], and followed by a larger outbreak in 2014 and 2015 in the Americas [4]. Although the symptoms of viral infection are usually mild and self – limiting [5], ZIKV infection is associated with severe neurological disorders including microcephaly in neonates and Guillain–Barré syndrome in adults, and may potentially also affect male fertility and cause testicular injury in mice [6–9], effects that have attracted global concern. Like other flaviviruses, Zika viruses are enveloped, positive-sense, single-standard RNA viruses [10]. The viral genome, 11 kb in length, encodes a single polyprotein that is subsequently cleaved into seven non-structural (NS) proteins, NS1, NS2A, NS2B, NS3, NS4A, NS4B, and NS5 and three structural proteins, namely, Capsid (C), premembrane/membrane (prM/M), and Envelope (E) proteins [11,12]. The NS proteins are mainly involved in the regulation of viral genome replication and immunological evasion [13–17], while structural proteins mainly help in assembling the virions, the swapping of the viral genome, and the packaging of the viral genome into progeny viruses [18]. The surface of mature viral particles is formed by 180 copies of E protein and M protein, in which E protein undergoes TRIM7-mediated polyubiquitylation at residues K38 and K281, leading to viral entry and pathogenesis [19].

Laminin receptor 1 (LAMR1), also known as ribosomal protein SA (RPSA), is a ubiquitously expressed, multifaceted protein [20]. Laminins are large heterotrimeric glycoproteins and the main structural components of the basement membrane [21]. LAMR1 is anchored at the cell membrane, where it regulates cell migration and invasion associated with metastatic cancers [22]. Recent studies have shown that LAMR1 is highly expressed in many cancer tissues and cells, including melanoma cells [23], pancreatic cancer cells [24], breast cancer cells [24], and esophageal cancer cells [25], and is associated with enhanced cell invasion and metastasis as well as prognosis [26,27]. In addition to the cell surface, LAMR1 is also found in the nucleus [28,29] and the cytoplasm [30], where it mediates development [31–33] and cell differentiation [32]. Moreover, LAMR1 is employed by pathogens as a receptor to trigger internalization [34] and promotes viral entry following infection by DENV, WNV, and JEV [35–37].

Interestingly, in this study, we demonstrate that LAMR1 is a host resistance factor that represses ZIKV infection. LAMR1 interacts with ZIKV E protein through its intracellular region (1–85aa), and attenuates the K48 – and K63-linked polyubiquitylation of E protein at both the K38 and K281 sites. Notably, we also found that LAMR1 promotes the deubiquitylation of E protein through recruiting eukaryotic translation initiation factor 3, subunit 5 (EIF3S5), a deubiquitination enzyme [38] reported to maintain the stability of STING through the removal of its K48-linked polyubiquitin chains [39]. We further found that the conserved E protein G282 residue is essential for LAMR1–E protein interaction and the LAMR1-induced deubiquitination of E protein. Collectively, these results demonstrated that LAMR1 interacts with ZIKV E protein and attenuates E protein ubiquitination through recruiting EIF3S5, resulting in the restriction of ZIKV infection.

## Results

### LAMR1 is a host restriction factor against ZIKV infection

**Figure 1.**
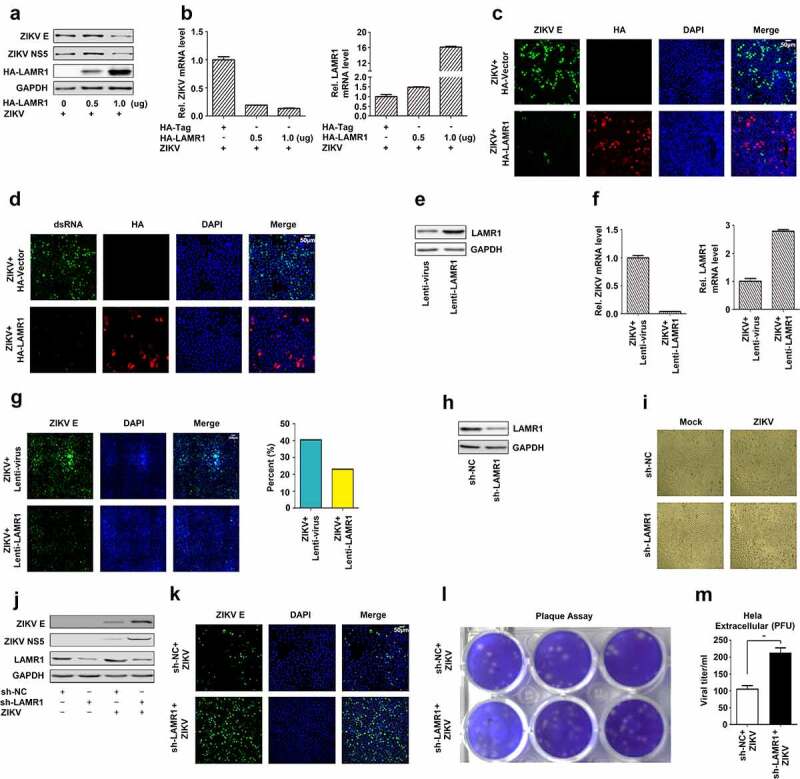
**LAMR1 is a host restriction factor against ZIKV infection**. (a–d) HeLa cells were transfected with pHA-LAMR1 or empty vector for 16 h and then infected with ZIKV (MOI = 1) for 48 h. The expression levels of ZIKV proteins were detected by immunoblotting (a) and confocal microscopy (c) and the viral RNA content was quantified by qPCR (b) and confocal microscopy (d). (e–g) Hela cells stably expressing LAMR1 or the control gene were generated and analyzed(e). Cells were infected with ZIKV (MOI = 1) for 48 h, following which viral RNA levels were quantified by qPCR (f) and ZIKV E protein levels by high-content analysis (g). (h–m) HeLa cells stably expressing sh-LAMR1 or control sh-RNA were generated and analyzed(h). Cells were infected with ZIKV (MOI = 1) for 48 h, and the cytopathic effects of cells was captured under the microscope (i). The expression levels of viral proteins were assessed by immunoblotting (j), while ZIKV titer in supernatants was calculated through a plaque assay (l, m)

LAMR1 was originally identified as a plasma membrane-localized receptor for laminins [20] and was shown to have multiple functions, including the mediation of infection by viruses, such as DENV [35], WNV [36], JEV [37] and Venezuelan equine encephalitis virus (VEEV) [40]. Here, we initially explored the role of LAMR1 in the regulation of ZIKV infection. Surprisingly, our results indicated that, in HeLa cells transfected with pHA-LAMR1 and infected with ZIKV, the production of ZIKV structural protein E and nonstructural protein 5 (NS5) was attenuated (Figure 1a), and the level of ZIKV RNA was significantly reduced (Figure 1b, left), as the level of LAMR1 mRNA increased (Figure 1b, right). Consistent with this, confocal microscopy analyses showed that LAMR1 overexpression led to a significant reduction in the levels of both ZIKV E protein (Figure 1c) and ZIKV dsRNA (Figure 1d). To further confirm the role of LAMR1 in the repression of ZIKV infection, HeLa cells stably expressing LAMR1 were generated by lentivirus infection (Figure 1e) and then infected with ZIKV. Compared with control cells, the abundance of ZIKV RNA was markedly reduced (figure 1f, left) in HeLa cells that stably expressed LAMR1 (figure 1f, right). In addition, immunofluorescence staining (Figure 1g, left) and fluorescence signal quantification (Figure 1g, right) using the Operetta high-content analysis system (Perkin–Elmer) confirmed that the content of ZIKV E protein was significantly downregulated in HeLa cells stably expressing LAMR1.

To determine the effect of eliminating LAMR1 expression on the repression of ZIKV infection, endogenous LAMR1 expression in HeLa cells was knocked down using LAMR1-specific shRNA (sh-LAMR1) (Figure 1h). In ZIKV-infected HeLa cells stably expressing sh-LAMR1, the cytopathic effects were markedly increased (Figure 1i), as were ZIKV E and NS5 protein levels (Figure 1j, k), and the level of ZIKV infection (Figure 1l, m). Collectively, these results demonstrated that the overexpression of LAMR1 leads to the repression of ZIKV protein production, mRNA expression, and viral infection, whereas knock down LAMR1 elicits the opposite effect. These results suggested that LAMR1 is a host restriction factor for ZIKV infection.

## LAMR1 binds to ZIKV E protein through its intracellular domain

**Figure 2.**
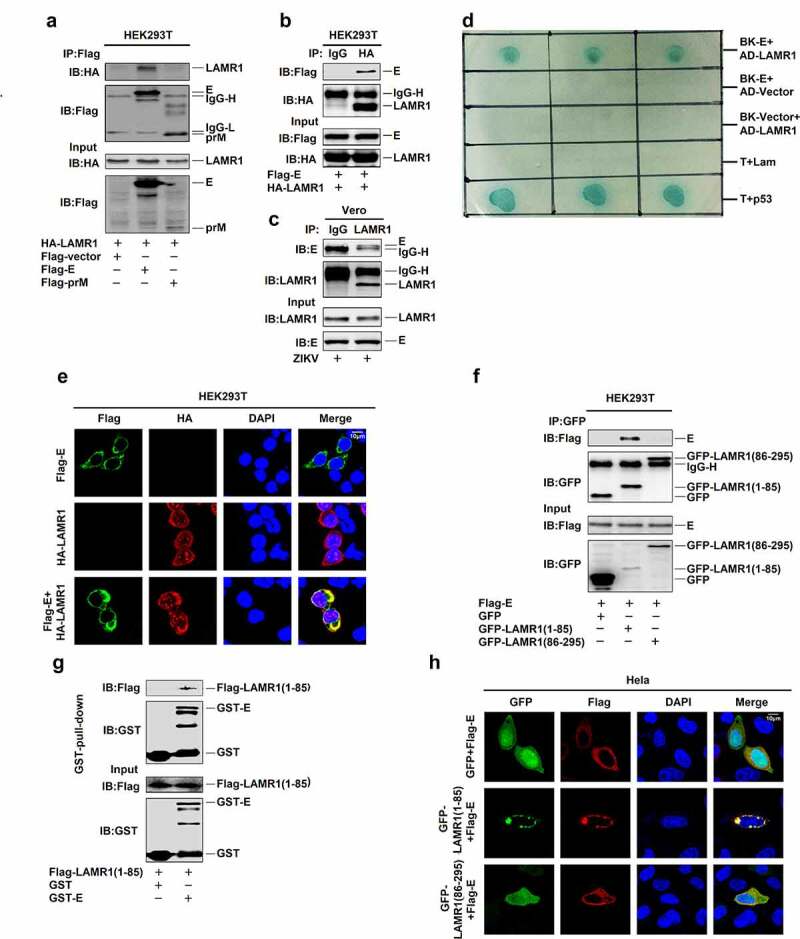
**LAMR1 binds to ZIKV E protein through its intracellular domain**. (a) HEK293T cells were transfected with plasmids encoding HA-LAMR1 and Flag-E or pFlag-prM. Cell lystes were prepared using lysis buffer and then used for immunoprecipitation (IP) with anti-Flag antibody and analyzed by SDS-PAGE. (b) HEK293T cells were transfected with plasmids encoding HA-LAMR1 and Flag-E. Cell lysates were prepared using lysis buffer and then used for IP with anti-Flag antibody or control IgG and analyzed by SDS-PAGE. (c) Vero cells were infected with ZIKV (MOI = 1) for 48 h and then subjected to IP with anti-LAMR1 antibody or control IgG. (d) A yeast two-hybrid screen was used to identify the interaction between LAMR1 and E protein. (e) HEK293T cells were transfected with pFlag-E or pHA-LAMR1, or co-transfected with pFlag-E and pHA-LAMR1. Immunofluorescence staining showed the sub-cellular localization of, HA-LAMR1 (red) and Flag-E (green); the nucleus is marked with DAPI (blue). (f) HEK293T cells were transfected with plasmids encoding Flag-E and GFP-vector/GFP-LAMR1 (1–85)/GFP-LAMR1 (86–295). Cell lysates were prepared using lysis buffer and then used for IP with anti-GFP antibody and analyzed by SDS-PAGE. (g) The Glutathione Sepharose beads were added to GST-E protein or GST only. The mixtures were then incubated with whole-cell extracts of HEK293T cells transfected with a plasmid encoding Flag-LAMR1 (1–85) (h) HEK293T cells were transfected with pFlag-E and pGFP-vector, pGFP-LAMR1 (1–85), and pGFP-LAMR1 (86–295). The sub-cellular localization of GFP, GFP-LAMR1 (1–85), GFP-LAMR1 (86–295) (green) and Flag-E (red) were analyzed by confocal microscopy; the nucleus is marked with DAPI (blue)

The mature flavivirus particle has an icosahedral surface consisting of 90 M protein-associated E protein dimers [41,42]. Here, we evaluated how LAMR1 exerts its antiviral effect on ZIKV. Co-immunoprecipitation (co-IP) results initially showed that LAMR1 interacted with ZIKV E protein, but not prM protein (Figure 2a) and further confirmed that E protein could interact with LAMR1 (Figure 2b). Notably, E protein co-immunoprecipitated with endogenous LAMR1 in ZIKV-infected Vero cells (Figure 2c). A yeast two-hybrid screen further confirmed the interaction between LAMR1 and ZIKV E protein (Figure 2d). Moreover, confocal microscopy indicated that ZIKV E protein was diffusely distributed outside the nucleus and that LAMR1 protein was diffusely distributed in the nucleus, cytoplasm and plasma membrane; meanwhile, LAMR1 and E proteins co-localization was observed in the cytoplasm and at the plasma membrane (Figure 2e).

A previous study reported that LAMR1 was a membrane-anchored protein containing a transmembrane domain residing within residues 86–106, an intracellular domain comprising residues 1–85, and an extracellular domain consisting of residues 107–295 [43]. Interestingly, co-IP assays showed that the intracellular region of LAMR1 (1–85aa), but not the transmembrane or extracellular region (87–295aa), could interact with ZIKV E protein (figure 2f). In Glutathione S-transferase (GST) pull-down assays, GST-E was pulled down with the LAMR1 intracellular region (residues 1–85) (Figure 2g). Confocal microscopy also showed that only the intracellular domain of LAMR1, but not the transmembrane or extracellular region co-localized with ZIKV E protein at the membrane (Figure 2h). These results demonstrated that LAMR1 can bind to ZIKV E protein through its intracellular domain.

## The conserved E protein G282 residue is essential for binding to LAMR1

**Figure 3.**
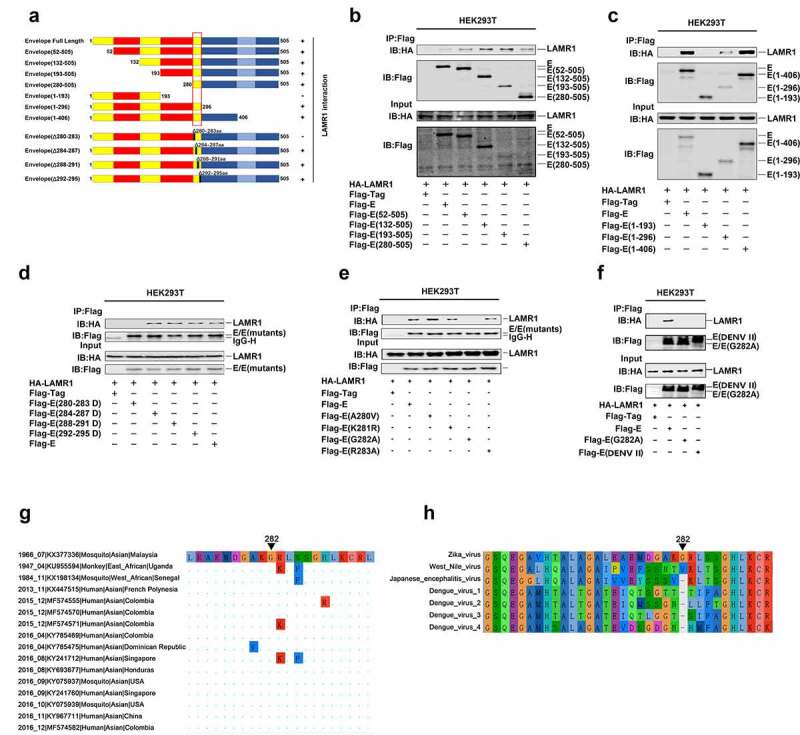
**The conserved E protein G282 residue is essential for the binding of LAMR1**. (a) Diagrams of the full-length E protein, truncated forms of E protein (1–505, 52–505, 132–505, 193–505, 280–505, 1–93, 1–296, and 1–406) and deletion forms of E protein (Δ280–283, Δ284–287, Δ288–291, and Δ292–295). (b) HEK293T cells were transfected with plasmids encoding HA-LAMR1 and Flag-vector, Flag-E/Flag-E (52–505), Flag-E (132–505), Flag-E (193–505), and Flag-E (280–505). Cell lysates were prepared using lysis buffer and then analyzed by immunoprecipitation with the indicated antibodies. (c) HEK293T cells were transfected with plasmids encoding HA-LAMR1 and pFlag-vector, Flag-E/Flag-E (1–193), Flag-E (1–296), and pFlag-E (1–406). Cell lysates were prepared using lysis buffer and then analyzed by immunoprecipitation with the indicated antibodies. (d) HEK293T cells were transfected with plasmids encoding HA-LAMR1 and Flag-vector, Flag-E, Flag-E (280–283aa deletion), Flag-E (284–287aa deletion), Flag-E (288–291aa deletion), and Flag-E (292–295aa deletion). Cell lysates were prepared using lysis buffer and then analyzed by immunoprecipitation with the indicated antibodies. (e) HEK293T cells were transfected with plasmids encoding HA-LAMR1 and Flag-vector, Flag-E, Flag-E G282A, and Flag-E DENV II. Cell lysates were prepared with lysis buffer and then analyzed by immunoprecipitation with indicated antibodies. (f) HEK293T cells were transfected with plasmids encoding HA-LAMR1 and Flag-vector, Flag-E, Flag-E A280V, Flag-E K281R, Flag-E G282A, and Flag-E R283A. Cell lysates were prepared with lysis buffer and then analyzed by immunoprecipitation with indicated antibodies. (g) Diagram of the consered G282 site. Viral sequences were downloaded from GenBank, and viewed and aligned using AliView software. (h) The diagrams of amino acid sequences of partial E protein among ZIKV, WNV, JEV and DENV. Sequences were downloaded from GenBank and viewed and aligned using AliView software

Structural analysis of ZIKV E protein has revealed that it contains three domains, including a central β-barrel like domain (domain I), an elongated finger-like structure (domain II), and an immunoglobulin-like module (domain III) [44]. To determine which specific region of E protein is required for its interaction with LAMR1, we constructed plasmids encoding the full-length E protein (1–505aa) and its truncated or mutant forms (Figure 3a). We found that, in addition to the full-length protein (1–505aa), four truncated forms, E (52–505aa), E (132–505aa), E (193–505aa), and E (280–505aa), could also interact with LAMR1 (Figure 3b). Moreover, two truncated forms of E protein, namely E (1–296aa) and E (1–406aa), could also interact with LAMR1; however, E (1-193aa) did not interact with LAMR1 (Figure 3c), indicating that the region of E protein containing residues 280–296 is involved in LAMR1 interactions. Three other E protein deletion mutants E (284–287 deletion), E (288–291 deletion), and E (293–295 deletion) could also interact with LAMR1, whereas E (280–283 deletion) could not (Figure 3d), suggesting that the residues 280, 281, 282, and 283 were important for the binding of E protein to LAMR1. We further found that three mutant forms of E protein with single aa substitutions at 280, 281, and 283 could interact with LAMR1, while E protein bearing a substitution at residue 282 did not associate with LAMR1 (Figure 3e). This indicated that amino acid G282 of E protein is essential for its binding to LAMR1.

Next, we performed a sequence alignment with several flaviviruses to evaluate the conservativeness of residue G282. Remarkably, the results showed that G282 is highly conserved among all ZIKV genome sequences dated from 1947 to 2017 and collected worldwide (only 16 ZIKV genome sequences are shown in this study) (Figure 3g), but not in the genomic sequences of WNV, JEV or DENV (Figure 3h). An additional co-IP assay also confirmed that LAMR1 didn’t interact with E protein with G282 substitution or E protein of DENV II that lacking G282 residue (figure 3f). Collectively, these results identified that the conserved residue G282 in ZIKV E protein is essential for its binding to LAMR1.

## LAMR1 attenuates K48 – and K63-linked E protein ubiquitination

**Figure 4.**
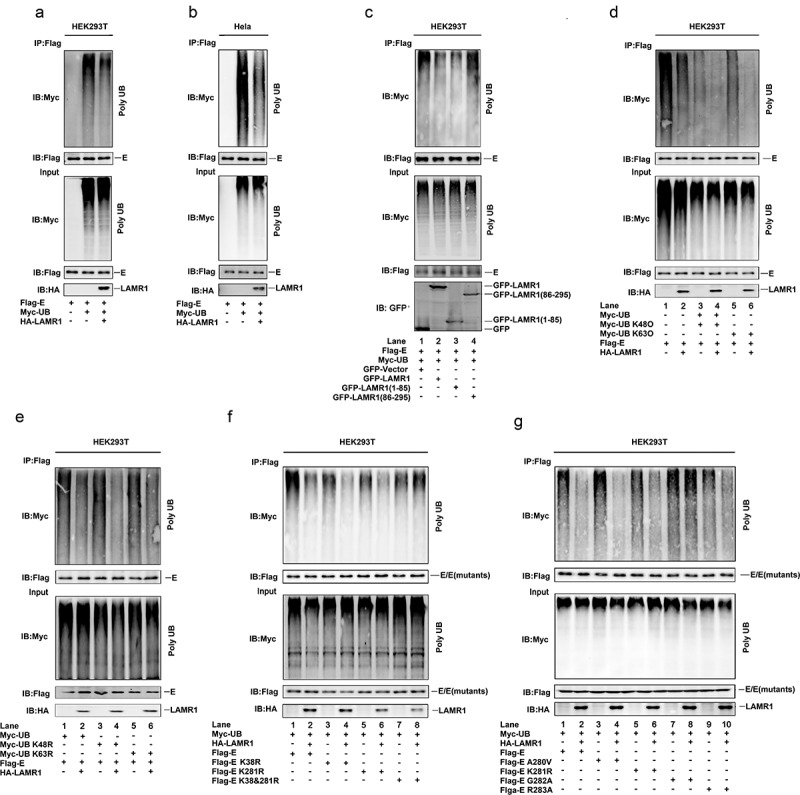
**LAMR1 attenuates K48 – and K63-linked E protein polyubiquitination**. (a, b) HEK293T cells and HeLa cells were co-transfected with pFlag-E, pMyc-UB, and pHA-LAMR1. Lysates were prepared and used for IP with the indicated antibodies and analyzed by SDS-PAGE. (c) HEK293T cells were transfected with pFlag-E, pMyc-UB and GFP, GFP-LAMR1, GFP-LAMR1 (1–85) and GFP-LAMR1 (86–295). Lysates were prepared and used for IP with the indicated antibodies and analyzed by SDS-PAGE. (d, e) HEK293T cells were co-transfected with pFlag-E, pMyc-UB, pMyc-UB K48O, pMyc-UB K63O, pMyc-UB K48R, Myc-UB K63R and HA-LAMR1. Lysates were prepared and used for IP with indicated antibodies and analyzed by SDS-PAGE. (f, g) HEK293T cells were co-transfected with pFlag-E, pFlag-E K38R, pFlag-E K281R, pFlag-E K38&281R, pFlag-E A280V, pFlag-E G282A, pFlag-E R283A, Myc-UB and HA-LAMR1. Lysates were prepared and used for IP with the indicated antibodies and analyzed by SDS-PAGE

Because a recent study reported that ZIKV E protein is polyubiquitinated at K38 and K281 residues during virus exocytosis, which drives viral entry and pathogenesis [19], and as our results showed that LAMR1 binds to ZIKV E protein, we then evaluated whether LAMR1 plays a role in the regulation of E protein ubiquitination. We found that the level of E protein ubiquitination was attenuated in both HEK293T cells and HeLa cells expressing exogenous LAMR1 (Figure 4a, b). We further noticed that only the intracellular region (1–85aa) of LAMR1 (Figure 4c, lanes 1–3), but not the region comprising the transmembrane and extracellular domains (86–295aa) (Figure 4c, lanes 1, 2 and 4), could promote E protein deubiquitination. Moreover, E protein polyubiquitination catalyzed by Myc-UB (Figure 4d, lanes 1 and 2), Myc-UB K48O (all lysine residues are mutated except K48) (Figure 4d, lanes 3 and 4) or Myc-UB K63O (all lysine residues are mutated except K63) (Figure 4d, lanes 5 and 6) was inhibited by LAMR1. Similarly, ZIKV E protein polyubiquitination catalyzed by Myc-UB (Figure 4e, lanes 1 and 2), Myc-UB K48R (only K63 is mutated) (Figure 4e, lanes 3 and 4) and Myc-UB K63R (only K63 is mutated) (Figure 4e, lanes 5 and 6) were suppressed by LAMR1. Overall, these results demonstrated that LAMR1 attenuates K48 – and K63-linked ZIKV E protein polyubiquitination.

Next, we generated three E protein mutants (K38R, K281R, and K38&281R) to investigate the specific E protein ubiquitination sites. Interestingly, LAMR1 attenuated E protein (figure 4f, lanes 1 and 2), E protein K38R (figure 4f, lanes 3 and 4) and E protein K281R ubiquitination (figure 4f, lanes 5 and 6) but not that E protein K38&K281R (figure 4f, lanes 7 and 8), indicating that LAMR1 removes E protein ubiquitination at both K38 and K281. As we found the conserved E protein G282 residue is essential for LAMR1–E interaction, we further evaluated whether this residue is involved in LAMR1-mediated E protein deubiquitination. We found that LAMR1 markedly reduced the ubiquitination levels of E protein (Figure 4g, lanes 1 and 2), E protein A280V (Figure 4g, lanes 3 and 4), E protein K281R (Figure 4g, lanes 5 and 6), and E protein R283A (Figure 4g, lanes 9 and 10), but not that of E protein containing the G282A substitution (Figure 4g, lanes 7 and 8). Collectively, these results suggested that LAMR1 attenuates K48 – and K63-linked ZIKV E protein ubiquitination at residues K38 and K281 in a protein–protein interaction-dependent manner.

## LAMR1 recruits EIF3S5 to deubiquitinate E protein

**Figure 5.**
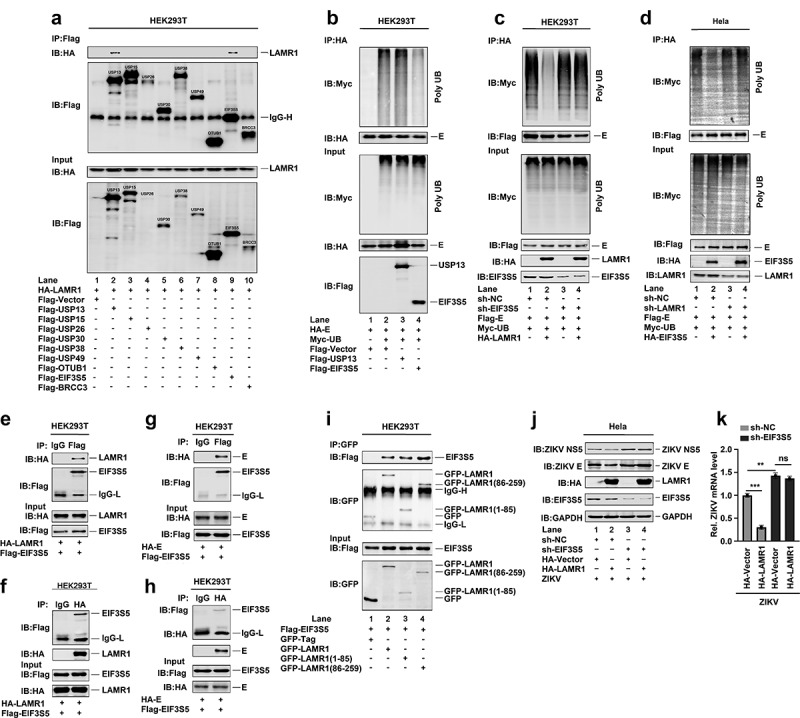
**LAMR1 recruits EIF3S5 to deubiquitinate ZIKV E protein**. (a) HEK293T cells were co-transfected with pHA-LAMR1 and pFlag-USP13, pFlag-USP15, pFlag-USP26, pFlag-USP30, pFlag-USP38, pFlag-USP49, pFlag-OTUB1, pFlag-EIF3S5, or pFlag-BRCC3. Lysates were prepared and used for IP with an anti-Flag antibody and analyzed by SDS-PAGE. (b) HEK293T cells were co-transfected with pHA-E, pMyc-UB, pFlag-USP13, and pFlag-EIF3S5. Lysates were prepared and used for IP with an anti-HA antibody and then analyzed by SDS-PAGE. (c) EIF3S5-knockdown HEK293T cells and control cells were co-transfected with pFlag-E, pMyc-UB, and pHA-LAMR1. Lysates were prepared and used for IP with an anti-Flag antibody and then analyzed by SDS-PAGE. (d) LAMR1-knockdown HeLa cells and control cells were co-transfected with pFlag-E, pMyc-UB, and pHA-EIF3S5. Lysates were prepared and used for IP with an anti-Flag antibody and then analyzed by SDS-PAGE. (e, f) HEK293T cells were transfected with plasmids encoding Flag-EIF3S5 and HA-LAMR1. Cell lysates were prepared with lysis buffer and then analyzed by IP with the indicated antibodies. (g, h) HEK293T cells were transfected with plasmids encoding Flag-EIF3S5 and HA-E. Cell lysates were prepared with lysis buffer and then analyzed by IP with indicated antibodies and immunoblotting as described above. (i) HEK293T cells were transfected with plasmids encoding Flag-EIF3S5 and GFP-LAMR1, GFP-LAMR1 (1–85aa), and GFP-LAMR1 (86–259aa). Cell lysates were prepared with lysis buffer and then analyzed by IP with the indicated antibodies. (j, k) HeLa cells stably expressing sh-EIF3S5 or control sh-RNA were generated and analyzed. Cells were transfected with pHA-LAMR1 or empty vector for 16 h, and then infected with ZIKV (MOI = 1) for 48 h. The levels of viral protein and RNA were detected by immunoblotting and qPCR, respectively

Next, we attempted to identify which enzyme of the ubiquitin proteasome system (UPS) was required for LAMR1-mediated E protein deubiquitination. For this, we screened several UPS enzymes, including UPS13, UPS15, UPS26, UPS30, UPS38, UPS49, OTU domain-containing ubiquitin aldehyde-binding protein 1 (OTUB1), eukaryotic translation initiation factor 3 subunit 5 (EIF3S5) and BRCA1/BRCA2-containing complex subunit 3 (BRCC3). We found that LAMR1 could interact with UPS13 (Figure 5a, lane 2) and EIF3S5 (Figure 5a, lane 9), but not with the control (Figure 5a, lane 1) or other proteins (Figure 5a, lanes 3–8, and 10), suggesting that EIF3S5 and UPS13 might be involved in the LAMR1-mediated deubiquitination of E protein. We further found that the level of E protein ubiquitination was attenuated in the presence of EIF3S5 (Figure 5b, lane 4), but not USP13 (Figure 5b, lane 3), indicating that EIF3S5 is involved in E protein deubiquitination. Notably, LAMR1 did not reduce the E protein ubiquitination level in HEK293T cells where EIF3S5 was knocked down (Figure 5c, lanes 3 and 4), confirming that EIF3S5 is essential for LAMR1-mediated E protein deubiquitination. Meanwhile, EIF3S5 cannot promote E protein deubiquitination in LAMR1-knockdown HeLa cells (Figure 5d), highlighting that LAMR1 recruits EIF3S5 to mediate E protein deubiquitination. Reciprocal co-IP assays further showed that LAMR1 and EIF3S5 interacted with each other in HEK293T cells (Figure 5e, f). Similarly, reciprocal co-IP assays also indicated that E protein and EIF3S5 protein were also interacted with each other in HEK293T cells (Figure 5g, h). Interestingly, as observed for full-length LAMR1 (Figure 5i, lane 2), both the intracellular region (1–85aa) (Figure 5i, lane 3) and the region comprising the transmembrane and extracellular domains (86–259aa) of LAMR1 (Figure 5i, lane 4) could interact with EIF3S5.

To evaluate the effect of EIF3S5 on LAMR1-mediated repression of ZIKV replication, we generated an EIF3S5-knockdown HeLa cell line. We found that ZIKV NS5 and E protein levels were reduced in the presence of LAMR1 in control HeLa cells (Figure 5j, lane 2 *vs*. lane 1), increased in EIF3S5-knockdown cells when compared with that in control cells (Figure 5j, lane 3 *vs*. lane 1), and not affected in EIF3S5-knockdown Hela cells in the presence of LAMR1 (Figure 5j, lane 4 *vs*. lane 3). Similarly, the level of ZIKV RNA was significantly reduced in the presence of LAMR1 in control HeLa cells, increased in EIF3S5-knockdown cells when compared with that in control cells, and not affected in EIF3S5-knockdown HeLa cells in the presence of LAMR1 (Figure 5k). Collectively, these results suggested that LAMR1 recruited EIF3S5 to deubiquitinate ZIKV E protein, thereby suppressing ZIKV replication and infection.

## Discussion

The prevalence of ZIKV has become a global public health concern owing to its association with devastating neurological complications [7,8] and male infertility [45,46]. Host innate immunity is the first line of defense against invasive pathogens while intrinsic factors also play roles in the defense against viral infections [47–49]. In this study, we demonstrated that LAMR1, originally identified as a laminin receptor [20], is a novel host restriction factor against ZIKV infection. The over-expression of LAMR1 leads to the repression of ZIKV infection, while the knock-down of endogenous LAMR1 elicits the opposite effect. Evaluation of the mechanism by which LAMR1 suppresses ZIKV infection revealed that LAMR1 protein binds to E protein with its intracellular region (1–85 aa), but not the transmembrane and extracellular region, indicating that LAMR1 is not involved in the regulation of viral entry into host cells. Additionally, a partial region of domain II (280–296aa) of ZIKV E protein contributes to LAMR1–E interaction. Notably, residue deletion and substitution analysis identified the essential role of E protein residue G282 in LAMR1–E interaction. Interestingly, we noted that residue G282 is highly conserved among known ZIKV genomic sequences but differs from that of other *Flavivirus* members, including DENV, WNV, and JEV. These data partly explain the different functions of LAMR1 in the regulation of *Flavivirus* infections, i.e., LAMR1 represses ZIKV infection, as demonstrated in this work, but promotes those of DENV, WNV, and JEV, as previously reported [35–37].

Ubiquitination is an important protein modification, regulating protein stability and function [50]. Ubiquitination system is reportedly required for flavivirus replication [51,52]. Moreover, the ZIKV infectious virion carries ubiquitinated E protein, which is polyubiquitinated during viral exocytosis and contributes to virus entry and pathogenesis [19]. Because we found that LAMR1 interacts with E through its intracellular region, we further evaluated the effect of LAMR1 on the ubiquitination level of E protein. Notably, our results showed that LAMR1 can attenuate K48 – and K63-linked E protein ubiquitination at both the K38 and K281 residues, two key E protein ubiquitination sites [19]; however, a recent study reported that E protein in the mature ZIKV virion is characterized K63-linked, but not K48-linked polyubiquitination, suggesting that the effects of attenuating K48-linked polyubiquitination by LAMR1 require further investigation. Moreover, E protein bearing the G282A substitution failed to interact with LAMR1, and this substitution abolished LAMR1-mediated deubiquitination of E protein, indicating that LAMR1 attenuates E protein ubiquitination in an interaction-dependent manner. Although we highlight that the substitution in the conserved G282 residue of E abolishes its binding to LAMR1 and inhibits LAMR1-promoted E protein deubiquitination, whether this substitution introduced in the virus could affect the ability of LAMR1 to restrict ZIKV infection needs confirmation through the reverse genetics approaches. Interestingly, we showed that EIF3S5 interacts with both LAMR1 and E protein, and further demonstrated that EIF3S5 is the deubiquitination enzyme required for LAMR1-mediated ZIKV E protein deubiquitination as well as the antiviral effects of LAMR1 against ZIKV infection.

In conclusion, in this study, we reported that LAMR1 is a novel host restriction factor against ZIKV infection. We also revealed a unique mechanism by which LAMR1 restricts ZIKV infection through attenuating E protein ubiquitination, which suggests that E protein ubiquitination may be a suitable target for the design of drugs to treat ZIKV infection. Moreover, our results showed that the intracellular region (1–85aa) of LAMR1 contributes to its binding to E protein and promotes E protein deubiquitination, suggesting that this region has the potential for use as a polypeptide drug for the treatment and prevention of ZIKV infection.

## Material and methods

### Cell lines and culture

HEK293T cells, HeLa cells, and C6/36 cells were purchased from American Type Culture Collection (ATCC, Manassas, VA, USA). HEK293T cells and HeLa cells were cultured in Dulbecco’s modified Eagle’s medium (DMEM) (Gibco, Grand Island, NY, USA), supplemented with 10% FBS, 100 U/mL penicillin, and 100 μg/mL streptomycin sulfate at 37^°C^ and with 5% CO_2_. C6/36 cells were cultured in RPMI 1640 medium (Gibco) supplemented with 10% FBS, 100 U/mL penicillin and 100 μg/mL streptomycin sulfate at 30°C with 5% CO_2_.

## Reagents

Antibodies against Flag (F3165), HA (H6908), and GAPDH (G9295) were purchased from Sigma (Darmstadt, Germany). The Anti-GFP antibody (66,002-1-Ig) was purchased from Proteintech (Wuhan, China). Antibodies against ZIKV NS5 protein (GTX133312) and E protein (GTX133314) were purchased from GeneTex (Hsinchu, Taiwan, P.R.C). Antibodies targeting LAMR1 were purchased from Abcam (Massachusetts, US) (ab133645) and ProteinTech (14,533-1-AP). The antibody against EIF3S5 (A7023) was purchased from Abclonal (Wuhan, China).

## Viruses

The ZIKV strain z16006 (GenBank accession number, KU955589.1) isolated by the Institute of Pathogenic Microbiology, Center for Disease Control and Prevention of Guangdong (Guangzhou, China) was used in this study.

## Plasmid construction

The cDNA fragment corresponding to ZIKV E protein and DENV II E protein was cloned into the C-terminal Flag-tagged pcDNA3.1 vector. Plasmids expression truncated E proteins (Envelope 52–505, Envelope 132–505, Envelope 193–505, Envelope 280–505, Envelope 1–93, Envelope 1–296, and Envelope 1–406), E protein deletion fragments (Envelope Δ280–283, Envelope Δ284–287, Envelope Δ288–291, and Envelope Δ292–295), and mutant E proteins (Envelope K38R, Envelope K281R, Envelope K38&281R, Envelope A280V, Envelope G282A, and Envelope R283A) were constructed by cloning the corresponding E-protein encoding gene fragments into C-terminal Flag-tagged pcDNA3.1 vector. Mammalian plasmids expressing HA-tagged LAMR1, GFP-tagged LAMR1, and Flag-tagged EIF3S5 were constructed by standard molecular cloning methods using the appropriate cDNA templates. Expression plasmids containing the intracellular region (1–85aa) and transmembrane and extracellular regions (86–259aa) of LAMR1 were constructed using the pEGFP-C1 vector.

## Lentivirus production and infection

The pLKO.1 vector (Sigma) carrying shRNAs specific for LAMR1 and EIF3S5 and the pLenti CMV vector (Addgene) expressing LAMR1 were transfected into HEK293T cells together with psPAX2 and pMD2.G using Lipofectamine 2000. Lentiviral particles were harvested at 36 and 60 h after transfection and then used to infect the indicated cells for 24 h with 4 μg/mL polybrene (Sigma). Positive cells were selected with 1.5 μg/mL puromycin (Sigma) for 5 days and detected by immunoblot analysis. The sequences of the primers for the shRNAs were as follows: Human sh-LAMR1: 5ʹ-CCTGCTGATGTCAGTGTTATA-3ʹ and Human sh-EIF3S5: 5ʹ-CTCTCAAGTGACTTGCAGCAA-3ʹ.

## Immunoblotting and immunoprecipitation

Cells were lysed with lysis buffer (150 mM NaCl, 50 mM Tris-HCl, 5 mM EDTA, 10% glycerol, 1% Triton X-100, and 1% protease inhibitor cocktail) (Roche). Western blot analysis was performed using the indicated antibodies. For immunoprecipitation, cell lysates were incubated with IgG or the indicated antibodies at 4°C overnight, after which protein A/G agarose (Pierce) was added to the lysates to the capture antibody complexes. After washing three times with the lysis buffer, the precipitates were recovered and subjected to SDS-PAGE and detected by immunoblotting using the indicated antibodies.

## Quantitative real-time PCR

Total RNA was extracted from cells with the Ultrapure RNA kit (CWBIO, Beijing, P.R.C.), and then reverse transcribed into cDNA using HiScript II Reverse Transcriptase (Vazyme, Nanjing, P.R.C.). Quantitative real-time PCR was performed with the ChamQ SYBR Color qPCR Master Mix (Vazyme) in a LightCycler 480 Real-Time PCR System (Roche). The sequences of the primers were 5ʹ-GGTCAGCGTCCTCTCTAATAAACG-3ʹ(sense) and 5ʹ-GCACCCTAGTGTCCACTTTTTCC-3ʹ (antisense) for ZIKV and 5ʹ-GGAGCGAGATCCCTCCAAAAT-3ʹ (sense) and 5ʹ-GGCTGTTGTCATACTTCTCATGG-3ʹ (antisense) for human GAPDH.

## Yeast two-hybrid analysis

*Saccharomyces cerevisiae* strain AH109; the control vectors pGBKT7, pGBKT7‐lam, pGBKT7‐T pGADT7, and pGADT7‐p53; and other reagents used in this study were purchased from Clontech Laboratories. All experimental steps were performed in accordance with the Matchmaker Golden Yeast Two-Hybrid – System User Manual.

## GST pull-down assays

*Escherichia coli* BL21 cells were transfected with the plasmid pGEX6p-1-E and grown at 37°C. Isopropyl β-D-1-thiogalactopyranoside (IPTG) was added to the cultures at an OD_600_ 0.6–0.8, followed by incubation for an additional 6 h at 20°C. The GST and GST-E proteins were purified from *E. coli*. In the GST-pull-down assay, the GST and GST-E proteins were incubated with glutathione agarose beads (Novagen). After incubation, the beads were washed three times with phosphate-buffered saline (PBS). HEK293T cells were transfected with the plasmid encoding Flag-LAMR1 (1–85 aa) and then lysed in the lysis buffer. The beads were incubated with the cell lysates at 4°C for 4 h. The precipitates were washed three times with lysis buffer, boiled in 2 × SDS loading buffer, separated by 12% SDS-PAGE, and immunoblotted with anti-GST and anti-Flag antibodies.

## Confocal microscopy

Cells were fixed in 4% paraformaldehyde at room temperature for 15 min, permeabilized with 0.1% Triton X-100 in PBS for 5 min, and then blocked with 5% bovine serum albumin (BSA). Next, the cells were incubated with the indicated antibodies overnight at 4°C, and further stained with FITC – or DyLight649-conjugated secondary antibodies. Finally, the samples were analyzed by confocal microscopy (Olympus FV1000).

## Data Availability

The authors confirm that the data supporting the findings of this study are available within the article.
